# Non-pharmacological Behavior Management Techniques in Pediatric Dentistry: A Bibliometric Analysis

**DOI:** 10.7759/cureus.41329

**Published:** 2023-07-03

**Authors:** Farah Shehani A, Sujitha Ponraj, Kavitha Ramar, Victor Samuel A, Rajakumar S, Gayathri J

**Affiliations:** 1 Pediatric and Preventive Dentistry, SRM Kattankulathur Dental College, SRM Institute of Science & Technology, Chennai, IND

**Keywords:** non-pharmacological behavior, pediatric dentistry, non-pharmacological, behavior shaping, behavior modification, behavior management

## Abstract

As an emerging trend, non-pharmacological behavior management has gained immense research interest. By utilizing a bibliometric approach, this investigation aims to review the state of the non-pharmacological behavior management techniques research in pediatric dentistry. A Scopus search was done on non-pharmacological behavior management in pediatric dentistry, including literature from 1900 to 2022, using “Cited Reference Search” on 4/2/2022. After being screened, the articles were ranked according to the number of citations they had, and the publication year, authorship, contributing institutions, countries, article topic, study design, H index, and keywords were extracted. Out of the 1431 articles found during the literature search, the top 50 cited articles were used for analysis. The citation counts of the 50 selected articles varied from 163 to one, and their highest publications were in the years 2017 and 2019 (n = 7). Most studies were published in the United Kingdom (n = 10). The studies done in Australia and USA have the highest mean citation with the most significant contributions from the Department of Dental Medicine, Children's Hospital, Regional Medical Center, USA. The systematic review was the most frequent study design (n = 19). Among 110 unique keywords, dental anxiety (n = 11) was the most frequently used. This bibliometric analysis offers valuable details on the top 50 publications cited between 2006 and 2022. Although these methods have been employed for many years, only recently have they been the subject of significant scientific publications. It is hoped that this study will enable aspiring and seasoned researchers to envision and create potential future scenarios for interdisciplinary research collaborations on the use of non-pharmacological behavior management techniques in pediatric dentistry.

## Introduction and background

A child’s good behavior is not magic; it takes various skills of encouragement to help them through their distress. One of the main qualities of a dentist is to manage a child positively while satisfying their dental needs. Thereby instilling a positive dental attitude and good oral health in them [1]. Wright et al. (1975) defined behavior management as the method by which the dental health team treats a young patient effectively and efficiently while also fostering a healthy dental attitude [1,2]. The first national clinical guidelines on non-pharmacological behavior management techniques were published online by the Royal College of Surgeons (England) in 2002. Since then, a considerable amount of research has been conducted in this area [1,3]. To handle children with behavioral difficulties, recent technology, such as audio-visual, video games, smartphones, and augmented reality, can be used as an alternative to traditional methods [4].

Bibliometric research is a comprehensive overview that analyzes research productivity, publications, citation patterns, top journals, most productive writers, most prestigious institutions, predominate subjects, keyword co-occurrences, and country-author-journal couplings as shown in the bibliography through time immemorial.

While attempting to investigate the clinical potential of non-pharmacological behavior management techniques in pediatric dentistry, numerous research clusters worldwide encountered hindrances. Therefore, the objective of this bibliometric analysis was to thoroughly review the most referenced research advancements on non-pharmacological behavior management techniques in pediatric dentistry to give them access to standard articles in this field.

## Review

Bibliometric methodology

Data Source

Scopus database was searched for publications on “non-pharmacological behavior management technique in pediatric dentistry” from 1990 to 2022 through Microsoft Excel (csv.) format (Microsoft Corporation, Redmond, WA). Citations, abstracts, keywords, and certain other pertinent bibliographic data were obtained from the literature.

Data Extraction

On February 2, 2022, the aforementioned database was searched. Using Scopus's search interface, the following search term was used to find literature on non-pharmacological behavior management techniques in pediatric dentistry: "article title, abstract, keywords." The search phrases "behavior management," "behavior modification," "behavior shaping," "pediatric dentistry," and "non-pharmacological" were merged using the Boolean operators "or" and "and." The total duration of the research was three months. The initial title search and abstract screening were done over a period of 45 days, following which complete article screening was performed. At the end of three months, bibliometric analysis was performed using VOSviewer software (Centre for Science and Technology Studies, Leiden University, Leiden, The Netherlands).

Inclusion/Exclusion Criteria

**Figure 1:**
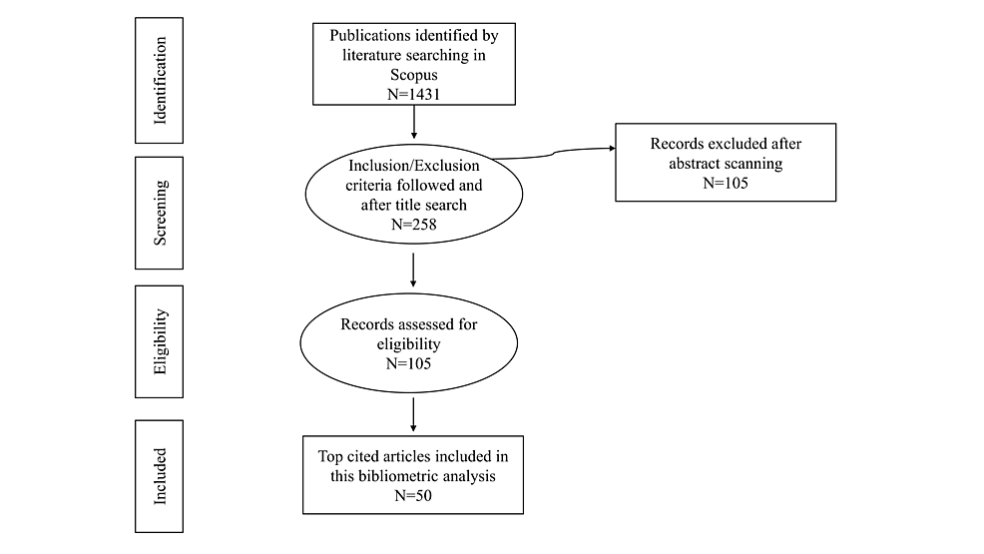
Flowchart of the review process

Comprehensive inclusion criteria included all types of studies on non-pharmacological behavior management techniques in pediatric dentistry. Only English-language articles were included. All articles published in general dentistry/medical departments with varied linguistic backgrounds were excluded. In the initial search, 1431 documents were found. Following the initial title search, which reduced the number of articles to 258, every record was then evaluated for appropriate and qualified articles, and ultimately, following the abstract search, the number was further reduced to 105. Amongst this, the top 50 cited articles were taken into this study. Selected records were published between 2006 and 2021. Three authors of this study repeated this strategy to be sure the data were accurate (Figure 1).

Data Analysis and Visualization

The top 50 selected documents and their related data were analyzed through Microsoft Excel (csv.) format and network visualization by VOSviewer software [4,5] for various factors.

Results

The top 50 cited publications on non-pharmacological behavior management techniques in pediatric dentistry were from 2006 to 2021. In view of the publishing and citation structure of non-pharmacological behavior management techniques in pediatric dentistry, the annual research output measured is shown in Table 1. It is clear that the first 10 articles on the subject were written between 2006 and 2008 and earned a total of 93 citations. Since then, there has been a gradual rise in research production. The second decade of the 21st century (2011-2022) saw substantial activity, with 44 publications released during that time (88% of the total 50 publications). In addition, these 44 papers received 497 citations in the same time frame (84% of all citations). Most publications (n = 7) were produced in the years 2017 and 2019. On the other side, the publication that was released in 2013 received the most citations (163) out of all of them.

**Table 1:** Leading countries in this research between 2006 and 2021 The annual research output in non-pharmacological behavior management technique, measured in terms of publication and citation count.

Top 24 countries	Country	Total publication	Total citation
1	United Kingdom	10	99
2	USA	7	209
3	Australia	2	180
4	Sweden	1	50
5	Philadelphia	1	31
6	Italy	4	63
7	China	4	28
8	Sharjah, UAE	1	14
9	Saudi Arabia	1	1
10	India	2	13
11	New Zealand	1	11
12	Canada	2	13
13	Germany	2	10
14	Singapore	1	6
15	Maharashtra	1	5
16	Turkey	1	1
17	Greece	1	5
18	Israel	1	5
19	Syria	1	3
20	Brazil	2	8
21	Serbia	1	1
22	Norway	1	2
23	Indonesia	1	1
24	Cuba	1	1

Type of Study

The records chosen for the bibliometric analysis included systematic review articles (n = 14), literature reviews (n = 9), narrative reviews (n = 2), randomized control trials (n = 10), case reports (n = 4), questionnaire studies (n = 6), retrospective studies (n = 1), comparative studies (n = 1), pilot studies (n = 1), and intervention studies (n = 1).

Leading Countries

**Figure 2:**
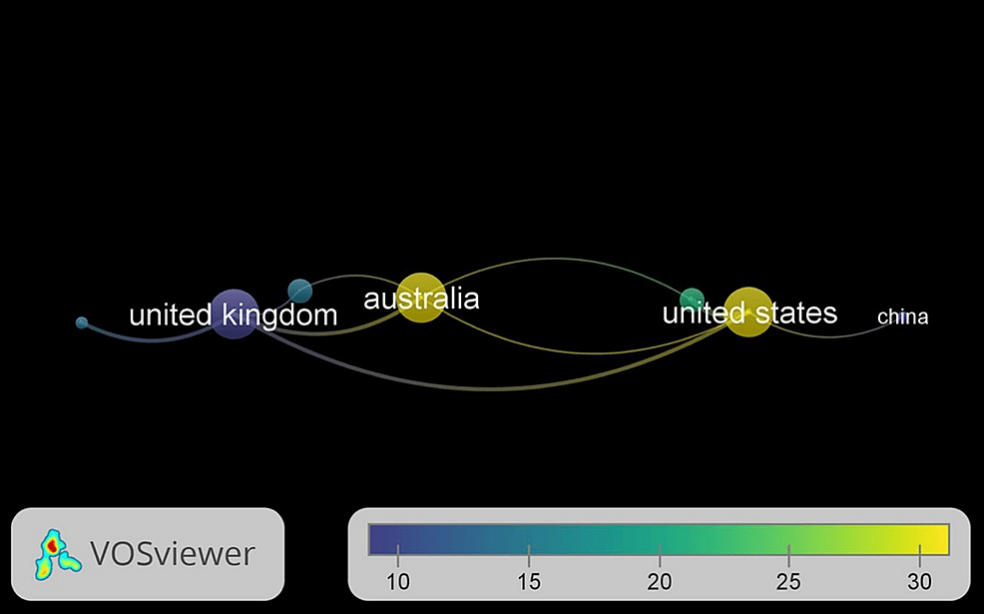
The nations acquiring the most citations.

Through analysis, it was established which nations from 2006 to 2021 had the most publications. These articles' citation impact and the number of citations received were also calculated, as indicated in Table 2 and depicted in Figure 2. The nations with the greatest publications were the UK (n = 10) [1,2], the USA (n = 7) [3], Italy (n = 4), and China (n = 4) [5]. The UK publications received 99 citations. Despite having fewer publications than the UK, the USA tops the list in terms of citations, with 209 citations for its seven publications. Australia only had two articles, but they received 180 citations, which is a high number.

**Table 2:** Leading organizations in this research between 2006 and 2021

S. No.	Top three organizations	Total publications	Total citations
1	The Regional Medical Center, Seattle, United States	2	84
2	Tufts University School of Dental Medicine, Boston, United States	2	209
3	Australian Research Centre for Population Oral Health at the University of Adelaide, Australia	2	180

Leading Institutions

The Regional Medical Center, Seattle, Washington, with the most publications (n = 2) and citations (n = 84) in non-pharmacological behavior management technique, was placed first among the top three leading institutions in this study (Table 2). Massey University in Wellington, New Zealand produced two articles that garnered 17 citations, and Lee Specialty Clinic in Louisville, Kentucky generated two publications that obtained two citations.

Most Successful Researchers

To determine the top four most constructive endeavors for the purposes of this study, the data were evaluated based on the sum of published works from various authors. To demonstrate their respective contributions to this field of study, the total citations, publications, and h-index were also calculated. Based on their citation scores, Table 3 lists the most prolific authors who have contributed to non-pharmacological behavior management research. Mancl L et al. (rank 1) from the University of Seattle (United States), Marshall J et al. from the Regional Medical Center (United States), Sheller B et al. from the University of Seattle (United States), and Williams BJ et al. from the Regional Medical Center (United States) in the scope of this study had the highest citation (n = 84) for their combined publication (n = 2), and their respective h-index is 10 each. Ashley PF et al. (rank 5) from the Military Dental Centre (Oman) had three publications with a citation score of 44 and an h-index of 11.

**Table 3:** List of authors with the highest number of citations in this research

Rank	Authors	Affiliations	Country	Total publications	Total citations​​​​​​​	H-index
1	Mancl L.	University of Seattle, United States	USA	2	84	10
2	Marshall J.	Regional Medical Center, United States	USA	2	84	10
3	Sheller B.	University of Seattle, United States	USA	2	84	10
4	Williams B.J.	Regional Medical Center, United States	USA	2	84	10
5	Ashley P.F.	Military Dental Centre, Oman	Oman	3	44	11
6	Facco E.	University of Padua, Italy	Italy	2	30	30
7	Zanette G.	University of Padua, Italy	Italy	2	30	30
8	Parekh S.	Majmaah University, Saudi Arabia	Saudi Arabia	2	27	11
9	Compton K.	Lee Specialty Clinic, United States	USA	2	2	10
10	Burson H.	New York University, United States	USA	2	2	10

Renowned Journals

The top 10 most popular journals, their publishers, country of origin, and impact factor, in non-pharmacological behavior management techniques in pediatric dentistry within the scope of this study are shown in Table 4. In terms of the top publications within the scope of this study, the International Journal of Pediatric Dentistry (n = 6), Pediatric Dentistry (n = 5), and European Archives of Pediatric Dentistry (n = 3) emerged as the top sources. The three highest impact factor journals, in descending order, are the Cochrane Database of Systematic Reviews (12.008), Journal of Pain Management (5.383), and Oral Diseases (4.068). The majority of the journals were from the UK.

**Table 4:** Top 10 most preferred journals in non-pharmacological behavior management techniques, their publishers, country of origin, and impact factor, in pediatric dentistry

S. No.	Journal	Publisher	Country	Impact factor
1.	International Journal of Pediatric Dentistry	Wiley-Blackwell Publishing Ltd	UK	3.264
2.	Pediatric Dentistry	Elsevier	USA	2.378
3.	Cochrane Database of Systematic Reviews	John Wiley & Sons Ltd	UK	12.008
4.	European Archives of Pediatric Dentistry	Springer	Germany	N/A
5.	Acta Odontologica Scandinavica	Taylor & Francis Group	Sweden	2.232
6.	Oral Diseases	Wiley-Blackwell	UK	4.068
7.	Child: Care, Health and Development	Wiley-Blackwell	UK	1.201
8.	Journal of International Dental and Medical Research	Ektodermal Displazi Grubu	Turkey	1.573
9.	Journal of Pain Management	Elsevier	USA	5.383
10.	Australian Dental Journal	Wiley-Blackwell	Australia	2.259

Keyword Co-occurrences

**Figure 3:**
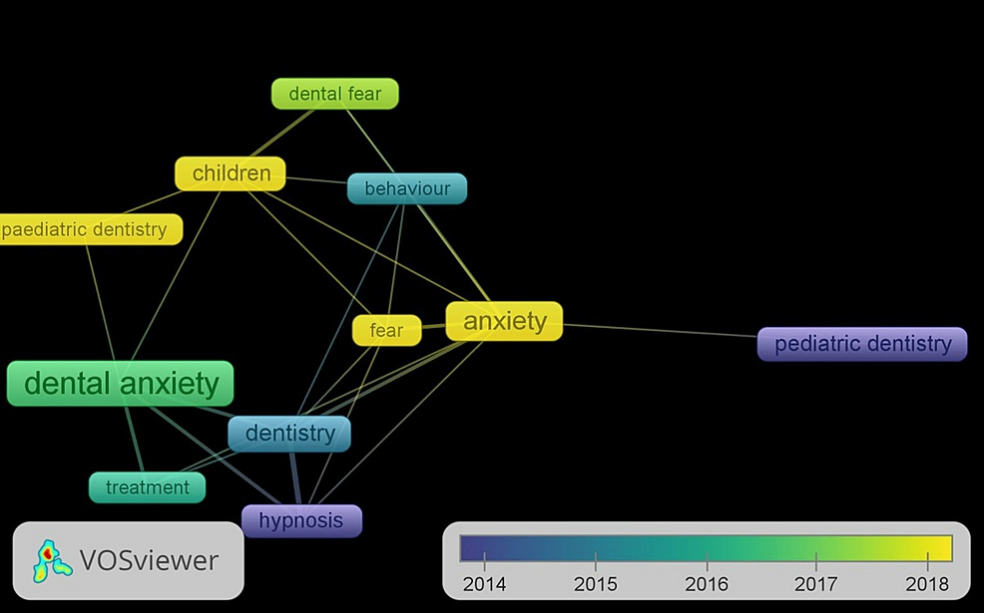
Keyword co-occurrences in this research

Figure 3 shows the keyword co-occurrences in non-pharmacological behavior management techniques in pediatric dentistry. A total of 110 keywords were examined, and those with three or more co-occurrences were considered. This standard was met by 12 keywords. The top five keywords, as shown in the graph below, were dental anxiety, anxiety, dentistry, children, and hypnosis, with corresponding weights of 11, 8, 6, and 4. Few co-occurrences [3] were found for the keywords "behavior," "fear," "dental fear," "behavior management," and "pediatric dentistry" in this group. Quantitative network indicators expressed the links as closely related keywords. The keyword with the highest frequency, dental anxiety, is connected to every other keyword.

Co-citation Network of Authors

**Figure 4:**
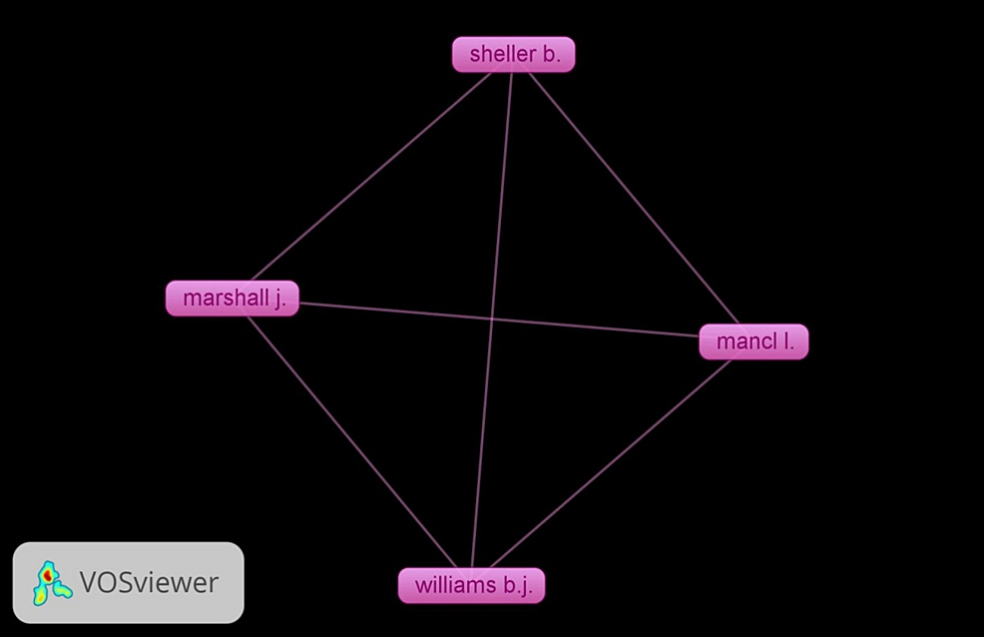
Co-citation network of authors in non-pharmacological behavior management techniques in pediatric dentistry between 2006 and 2022

Figure 4 shows the co-citation network of authors in non-pharmacological behavior management techniques in pediatric dentistry from 2006 to 2022. Seven authors meet the requirement out of the authors with 30 or more citations. The top four authors with a weightage (citations) of 84 each are Mancl L et al., Marshall J et al., Sheller B et al., and Williams BJ et al. Quantitative network indicators indicate authors.

Discussion

To our knowledge, this bibliometric analysis is the first of its type to identify and statistically analyze the top 50 referenced scientific research papers expressing the core of non-pharmacological behavior management techniques in the field of pediatric dentistry from 1900 to 2022. Hence, the decision to focus on the top 50 articles was to identify the most influential and impactful research publications in the field. By concentrating on the top-cited articles, we aimed to capture the seminal works that have shaped the current understanding and development of the subject matter. Hence, it allowed us to delve deeply into the core literature and extract meaningful insights.

In our research article, only VOSviewer software was employed for data analysis and visualization based on its well-established reputation and suitability for bibliometric analysis. VOSviewer offers powerful functionalities for analyzing citation networks, identifying key themes, and visualizing bibliographic data. Its user-friendly interface and flexibility made it an ideal choice for our research needs.

The yearly citation and publication analysis of the research articles in this study showed that the most highly referenced paper on non-pharmacological behavior management techniques was published in Australia in 2013. There have been 163 citations for it. The other noteworthy article was written by Loo CY et al. (USA) and appeared in the European Archives of Pediatric Dentistry. It has received 73 citations (2010). When protective stabilization cannot be achieved, sedation or general anesthesia is the preferred behavioral guidance tool, according to Loo CY et al. [6-10]. After 2007, the practice of publishing articles on non-pharmacological behavior management techniques in pediatric dentistry experienced an exponential rise in popularity.

From this study, the top four most productive nations were the UK, the USA, Italy, and China, where the UK was rich in a number of publications and Australia in a high number of citations. This result was achieved by the pediatric oral healthcare professionals' interest in improving the dentistry curriculum to dispel myths and encourage a thorough and optimistic perspective on issues with managing dental anxiety, dental fear, and dental behavior [8]. Furthermore, from the years 2017 to 2019, there has been an increase in the popularity of hypnosis [11]. This unique investigation was conducted in the UK by Dailey et al. [12]. The article was based on a postal survey that was sent to all 328 dentists whose names appeared in the British Society for Behavioural Sciences in Dentistry Directory asking them about the methods they used to assist worried dental patients [12]. As of now, there is no proof that dental professionals in Australia are using them. This knowledge gap is a challenge in tackling the pervasive dental phobia in the population [13]. The worry that chairside evaluations of patient anxiety and terror could be insufficient exacerbates the problem. Evidence from the USA suggests that a patient's self-reported anxiety using a variety of dental fear scales and a dentist's assessment of their anxiety have only a small-to-moderate association [14]. Hence, Armfield JM et al. sought to delve deeper into this issue [13]. The American Tufts University School of Dental Medicine actively supports dental research in both the public and private sectors. The US was ranked second, after the UK, in more publications and citations, possibly as a result of the country's very competitive residency program [10,14]. The Regional Medical Center in Seattle (USA) and the Australian Research Centre for Population Oral Health at the University of Adelaide in Australia were the two most prolific institutions. Additionally, the dominance of the USA in the list of most productive universities with the most citations may be explained by the existence of numerous research clusters across various institutions with a shared area of interest [15-18]. Because of this, five of the top 10 most referenced authors, including Mancl et al., Sheller et al., Marshall et al., and Williams BJ et al., were linked to the same American universities, Regional Medical Center and University of Seattle, respectively.

The International Journal of Pediatric Dentistry (n = 6), Pediatric Dentistry (n = 5), and European Archives of Pediatric Dentistry (n = 3) stood out as the top journals within the purview of this investigation. The top three journals were published in the US and the UK. This demonstrates the pattern in which industrialized nations have been significantly advancing this field of study. The three journals with the most significant impact factors are the Cochrane Database of Systematic Reviews (12.008), the Journal of Pain Management (5.383), and Oral Diseases (4.068). The majority of the journals were from the UK. Additionally, they exclusively publish papers in English, as was the case with the data chosen for this study.

In the context of this study, the papers selected for examination showed a distinct trend in the keywords. Typically, keyword co-occurrence networks are built using terms that have been retrieved from publication titles, abstracts, or even originator keyword lists [19]. If two keywords appear in the same abstract/title or main context, they are called to co-occur. Additionally, there is a nearly inverse relationship between the phrases' similarity and their separation. As a result, it is usually discovered that terms with a greater relevance are located closer to one another [20]. The top five most commonly used keywords were hypnosis, dental anxiety, anxiety, children, and dentistry. The keyword with the highest frequency, dental anxiety, is tied to all the other phrases. This is due to the significant number of cases of dental anxiety recorded [21].

Co-citation network analysis of authors, which is used in the bibliographic analysis, aids in examining the subjacent fetes in a field in terms of the groups of writers, where all these were referenced collectively in the pertinent history. It also gives details on the authors' methods, who are subject-matter experts, and views the connections between published works [22]. The authors of this study, i.e., Mancl L. et al. and Sheller B et al., are both listed as hailing from the University of Seattle in the USA, while Marshall J et al. and Williams BJ et al. [18] are both listed as hailing from the Regional Medical Center in the USA. We would hope to see more joint work with them in the near future, as they look to have dominated this field of research thus far.

Regarding the choice of Scopus database, we selected Scopus instead of Web of Science, PubMed, or Google Scholar databases, due to its extensive coverage of scientific literature across multiple disciplines, including the field of our research. Scopus is widely recognized for its comprehensive indexing, citation tracking, and access to high-quality research articles, to find author information, such as h-index, and lists of publications, and locate impact metrics for a journal title using SNIP (Source Normalized Impact per Paper), SJR (SCImago Journal Rank), and CiteScore. By utilizing Scopus, we aimed to ensure a robust and comprehensive review of the relevant literature in our study area.

Additionally, the self-citation of authors was not taken into account. Although in the context of the co-citation network of authors in non-pharmacological behavior management techniques in pediatric dentistry, citation density can be understood as the number of citations between specific authors or the overall density of citations within the network. From 2006 to 2022, the network analysis identified seven authors who met the requirement of having 30 or more citations. Among these authors, the top four, namely, Mancl L et al., Marshall J et al., Sheller B et al., and Williams BJ et al., have a weightage or citation count of 84 each. While citation density can be calculated more precisely with access to the specific citation data, we can make some observations based on the information provided. In this case, the four top authors mentioned have the same weightage of 84, indicating that they have received a similar number of citations. This suggests a relatively high citation density for these authors within the co-citation network. Their work has likely been influential and extensively cited by other authors in the field of non-pharmacological behavior management techniques in pediatric dentistry. In terms of their average citation score per publication, for Mancl L et al. from the University of Seattle, citation density = total citations/total publications, i.e., citation density = 84/2 = 42; for Marshall J et al. from the Regional Medical Center, citation density = total citations/total publications, i.e., citation density = 84/1 = 84; for Sheller B et al. from the University of Seattle, citation density = total citations/total publications, i.e., citation density = 84/1 = 84; for Williams BJ et al. from the Regional Medical Center, citation density = total citations/total publications, i.e., citation density = 84/1 = 84; for Ashley PF et al. from the Military Dental Centre, citation density = total citations/total publications, i.e., citation density = 44/3 = 14.67. Please note that the citation density calculated here represents the average number of citations per publication for these top researchers. As a limitation of this study, the citation density was not done for all the individual articles, hence this can be a perspective for a future study.

Beyond its limits, this study may help future researchers by pointing out potential directions for investigation and highlighting research gaps. It would assist them in identifying the most significant nations, colleges, writers, journals, and keywords while researching the subject and seeking partnerships, thereby increasing the number of publications in journals with high impact factors. Expanding the search to more databases and examining the clinical applications of emerging trends in non-pharmacological behavior management techniques, such as hypnosis [13,23] in pediatric dentistry, could be of interest to researchers in the future. By utilizing the body of research on the practical application of non-pharmacological behavior management techniques in pediatric dentistry, we can also make significant progress in modifying the guidelines.

## Conclusions

Analyzing the top 50 cited articles in non-pharmacological behavior management in pediatric dentistry can provide valuable insights. It identifies influential research, reveals trends and methodologies, assesses research impact, guides future research directions, informs evidence-based practice, and promotes collaborations in the field. This study is significant for understanding foundational research, identifying gaps, and advancing knowledge in non-pharmacological behavior management techniques for pediatric dental care.
